# Unusual Case of Neuromeningeal Late Relapse of POLE Mutated Endometrioid Carcinoma: A Case Report and Systematic Review

**DOI:** 10.3390/curroncol33040219

**Published:** 2026-04-16

**Authors:** Emma Donati, Michel Fabbro, Noémie Drappier, Alexis Marguerit, Cristina Leaha, Stéphanie Nougaret, Pierre-Emmanuel Colombo, Stanislas Quesada

**Affiliations:** 1 Department of Medical Oncology, Institut du Cancer de Montpellier (ICM), 34090 Montpellier, France; michel.fabbro@icm.unicancer.fr (M.F.); noemie.drappier@icm.unicancer.fr (N.D.); stanislas.quesada@icm.unicancer.fr (S.Q.); 2 Groupe d’Investigateurs Nationaux pour l’Etude des Cancers de l’Ovaire et du Sein (GINECO), 75008 Paris, France; pierre-emmanuel.colombo@icm.unicancer.fr; 3 Department of Radiation Oncology, Institut du Cancer de Montpellier (ICM), 34090 Montpellier, France; alexis.marguerit@icm.unicancer.fr; 4 Department of Pathology, Institut du Cancer de Montpellier (ICM), 34090 Montpellier, France; cristina.leaha@icm.unicancer.fr; 5 Department of Radiology, Institut du Cancer de Montpellier (ICM), 34090 Montpellier, France; stephanie.nougaret@icm.unicancer.fr; 6 Department of Surgical Oncology, Institut du Cancer de Montpellier (ICM), 34090 Montpellier, France

**Keywords:** endometrioid endometrial carcinoma, POLE mutation, molecular classification, brain metastasis, late recurrence

## Abstract

**Background**: POLE-mutated endometrial carcinomas are associated with exceptionally favorable outcomes, forming the basis for treatment de-escalation in early-stage disease. Nevertheless, rare adverse clinical courses have been reported. This study describes an unusual case of late metastatic recurrence in a POLE-mutated tumor and provides a review of similar cases in the literature. **Methods**: We present a detailed clinical, radiological, pathological, and molecular description of a patient who developed metastatic recurrence 16 years after initial surgery. A systematic literature search was conducted to identify reports of recurrence, progression, or cancer-related death in POLE-mutated endometrial carcinoma, with extraction of recurrence patterns, genomic features, treatment, and outcomes. **Results**: The patient experienced sequential pulmonary, cerebral, and leptomeningeal metastases despite harboring a canonical POLE hotspot mutation, proficient mismatch repair status, wild-type TP53, no additional known driver mutation beyond PTEN alterations. The literature review identified a small number of similarly adverse cases. Reported recurrences were heterogeneous, though distant and occasionally central nervous system involvement were noted. **Conclusions**: While POLE-mutated tumors overall retain an excellent prognosis, rare cases may follow an atypical and aggressive course. Improved molecular annotation and integrated risk-stratification models are needed to better identify this minority of higher-risk patients.

## Simple Summary

Endometrial carcinoma is the most common malignancy in high income countries. Among its different molecular subtypes, tumors with a POLE mutation are known to have particularly excellent outcomes, with very low risk of recurrence. However, rare exceptions exist. In this report, we describe the unusual case of a woman whose endometrioid endometrial carcinoma relapsed 16 years after her initial treatment, first in the lung and then in the brain. We also reviewed published cases showing similarly unexpected outcomes in this tumor subtype. Although these situations are extremely uncommon, they raise important questions about how best to follow patients over time and highlights the need for continued research to better identify the rare patients who may require closer monitoring or tailored treatment strategies.

## 1. Introduction

Endometrial cancer is the most common gynecological malignancy in high-income countries, with endometrioid adenocarcinoma representing the predominant histological subtype [1,2]. Advances in molecular classification, particularly those arising from The Cancer Genome Atlas (TCGA), have led to the identification of distinct molecular subgroups with different prognostic implications [3]. Among these, polymerase epsilon (POLE) ultramutated tumors are characterized by an exceptionally high mutation burden and are associated with excellent clinical outcomes, including very low recurrence rates.

To date, eleven pathogenic POLE mutations have been identified as “hotspot” variants. Five of these (P286R, V411L, S297F, A456P, and S459F) were initially described by TCGA as being most commonly associated with the ultramutated phenotype [3]. The remaining six were later validated using a pragmatic scoring algorithm, based on tumor mutational burden and clinicopathological features [4].

Robust survival data from PORTEC-1, PORTEC-2 and PORTEC-3 [5,6,7] further support the excellent prognosis of POLE-mutated endometrial cancers. These findings form the basis of current international guidelines recommending treatment de-escalation for stage I–II POLE-mutated endometrial cancers.

Within this overall favorable context, rare instances of recurrence and cancer-related death have nonetheless been documented in patients with POLE-mutated tumors. These exceptional cases do not challenge the overarching paradigm of excellent outcomes, but they raise important questions regarding potential biological heterogeneity within this subgroup, optimal long-term surveillance strategies, and the nature of the atypical—and exceedingly uncommon—recurrence patterns that may arise in POLE-mutated cancers.

In this manuscript, we describe an unusual case of a POLE-mutated endometrioid endometrial carcinoma (EEC) with an exceptionally late metastatic recurrence with subsequent leptomeningeal carcinomatosis, ultimately leading to cancer-related death. To contextualize this case within the existing literature, we conducted a systematic review of published reports describing adverse outcomes in POLE-mutated endometrial cancer, interpreted alongside the overwhelming data demonstrating excellent prognosis in the majority of POLE-mutated tumors.

## 2. Materials and Methods

### 2.1. Case Report Methodology

This case report was written in accordance with the CARE (CAse REport) guidelines to ensure transparent and comprehensive reporting [8]. Clinical, pathological, radiological, and molecular data were retrieved from the patient’s medical record with appropriate institutional permission. Imaging findings, treatments, and follow-up information were reviewed by the multidisciplinary team involved in patient care. Molecular analyses, including POLE sequencing and assessment of co-occurring alterations, were performed as part of routine diagnostic work-up at the time of recurrence.

### 2.2. Systematic Review Methodology and Rationale

This systematic review was conducted according to the Preferred Reporting Items for Systematic Reviews and Meta-Analysis (PRISMA) guidelines [9]. The PRISMA checklist is available as Supplementary Materials. Its purpose was not to reassess the overall prognosis of POLE-mutated endometrial carcinoma—already firmly established through randomized trials—but rather to identify and describe rare cases of POLE-mutated tumors with unfavorable clinical evolution in which detailed recurrence features were available.

Because large randomized trials provide robust survival outcomes but do not report granular data on extremely rare recurrences (e.g., timing, metastatic patterns, treatment at relapse, molecular annotation at recurrence), they were not included as part of the systematic search, but were examined separately to contextualize overall prognosis.

For instance, ten-year cancer-specific survival reached 100% for POLE-mutated tumors in PORTEC-1 and PORTEC-2 [5,6], and the 10-year recurrence-free survival was 98.0% in PORTEC-3 [7]. These data support guideline recommendations for treatment de-escalation, yet do not provide sufficient detail to characterize outlier cases.

A systematic search of PubMed was performed on 22 May 2025, using the following Medical Subject Heading (MeSH) terms: (endometrial neoplasms) AND (POLE protein, human. No restrictions were applied regarding publication date. Articles in English were eligible. References from selected studies were manually screened to identify additional publications. Because of the rarity of the event of interest, additional searches were performed during manuscript preparation to ensure completeness. This systematic review was not prospectively registered, given its exploratory nature and focus on rare reported cases.

All identified records were screened manually. Studies were eligible for full-text review if they met all of the following criteria:

- Reported cases of endometrioid endometrial carcinoma (EEC) with a sequencing-confirmed POLE mutation.
- Provided individual-level clinical information allowing extraction of recurrence timing, recurrence pattern, and/or treatment at relapse.
- Reported an unfavorable evolution, defined as recurrence, progression, metastasis, or cancer-related death.

Studies were excluded if they:

- Did not specify POLE mutation by sequencing or reported only favorable outcomes.
- Included non-endometrioid histology without separable data.
- Reported only variants of unknown significance.
- Were reviews, editorials, commentaries, or duplicate reports.

Because some older studies predated current molecular standards, all reported mutations were reassessed and classified as canonical (hotspot) or non-canonical according to contemporary criteria.

**Figure 1:**
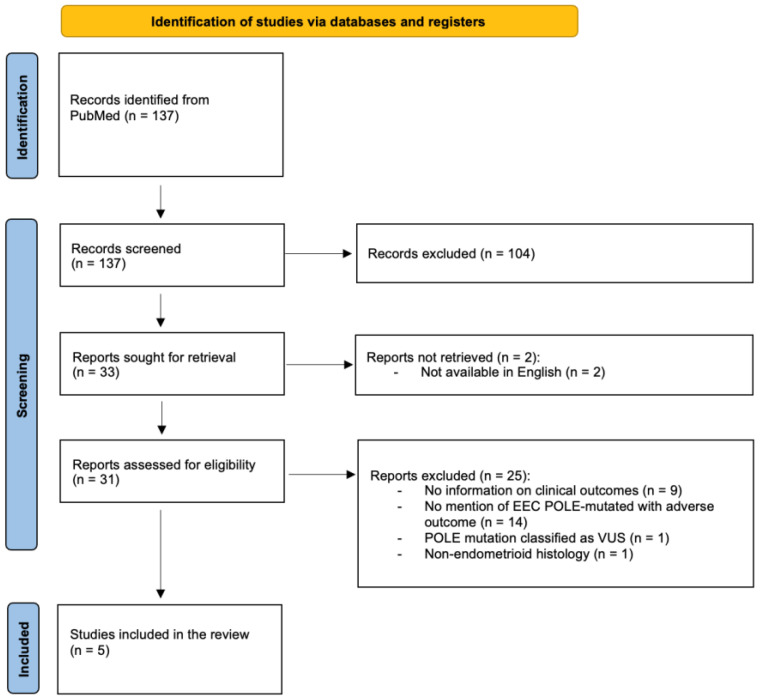
PRISMA flow diagram of the study selection process for the systematic review.

The study selection process is detailed in the PRISMA flow diagram provided as Figure 1.

The following variables were extracted whenever available:

- Patient characteristics,
- FIGO stage, histological grade,
- Type of POLE mutation (hotspot vs. non-hotspot),
- Mismatch repair (MMR) status and other co-alterations,
- Recurrence interval,
- Recurrence/metastatic site(s),
- Management and survival following relapse.

Given the expected heterogeneity in reporting, a qualitative, narrative synthesis was performed without quantitative meta-analysis.

## 3. Results

### 3.1. Case Report

In 2006, a 57-year-old postmenopausal woman, with a personal history of diabetes mellitus, hypercholesterolemia and overweight, initially presented to her gynecologist with post-menopausal metrorrhagia, leading to the diagnosis of an endometrial tumor. She underwent a total hysterectomy, bilateral salpingo-oophorectomy and pelvic lymphadenectomy. Histopathological analysis confirmed an endometrioid carcinoma, grade 2, staged as pT1bN0 according to FIGO stage IA (UICC 7th edition). At the time of initial diagnosis, the status of hormonal receptors, POLE, mismatch repair (MMR) and TP53 were unknown. The patient did not receive any adjuvant therapy.

**Figure 2:**
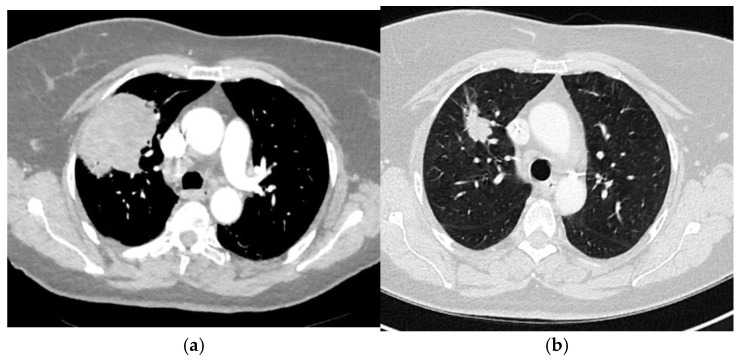
Axial computed tomography images showing an isolated pulmonary relapse: (**a**) before induction chemotherapy; (**b**) after induction chemotherapy.

The patient was then followed up for 16 years: 5 years in our center in accordance with institutional protocol and subsequently monitored by her general practitioner. Sixteen years after initial treatment, she presented with a persistent cough for 5 months, associated with a decline in general health including a 7 kg weight loss. A chest radiography revealed a right lung mass, subsequently confirmed by chest computed tomography (CT), which showed an isolated 7 cm upper right lung lesion (Figure 2a). Histopathological examination of the lung biopsy revealed a poorly differentiated carcinoma (solid growth with marked nuclear atypia and high mitotic activity) consistent with endometrial origin. Immunohistochemistry showed CK7 and PAX8 positivity, and negativity for TTF1 and neuroendocrine markers. Strong hormone receptor expression (ER and PR, 100%) and a high proliferation index (Ki-67 ~80%) were observed. These findings supported the diagnosis of metastatic endometrial carcinoma. Next-generation sequencing (NGS) identified a POLE p.(Pro286Arg) exon 9 mutation with an allelic frequency of 32.64%, and two PTEN mutations: p.(Tyr336*) exon 8 (allelic frequency 29.71%) and p.(Ser59*) exon 3 (allelic frequency 29.4%), with no TP53 mutation identified. The subsequent metastatic follow-up, including a PET-CT scan, did not reveal any other secondary lesions.

The patient received induction chemotherapy with six cycles of carboplatin plus paclitaxel every 3 weeks. Reevaluation after 6 cycles demonstrated a partial response, with the pulmonary lobar mass decreasing from 73 mm to 31 mm (Figure 2b). Subsequently, the patient underwent robot-assisted right upper lobectomy, which was deemed complete (R0). Histopathological analysis of the resected lobe confirmed a metastasis of the previously diagnosed endometrioid carcinoma, showing high-grade features.

**Figure 3:**
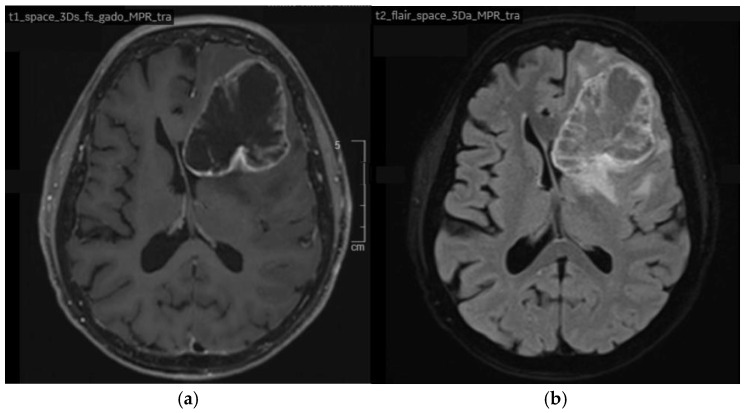
Axial brain magnetic resonance imaging (MRI) showing an isolated left frontal metastasis: (**a**) T1-weighted sequence after gadolinium administration; (**b**) T2-weighted FLAIR sequence.

Following a period of active surveillance of three months, the patient developed progressive neurological symptoms, including cognitive impairment and rapidly worsening amnesia. Brain MRI performed shortly after revealed a new solitary 62 mm left frontal lesion (Figure 3a,b), which exerted a mass effect on medial structures, with an 8 mm subfalcine herniation.

Neurosurgical intervention was proposed, and the patient underwent surgery. Histopathological analysis confirmed findings consistent with a metastasis of her known endometrioid carcinoma. Adjuvant cerebral radiotherapy to the surgical bed was administered, delivering a dose of 30 Gy in 10 fractions of 3 Gy using Volumetric Modulated Arc Therapy (VMAT). Given the confirmed hormonal sensitivity, hormone therapy was then initiated.

**Figure 4:**
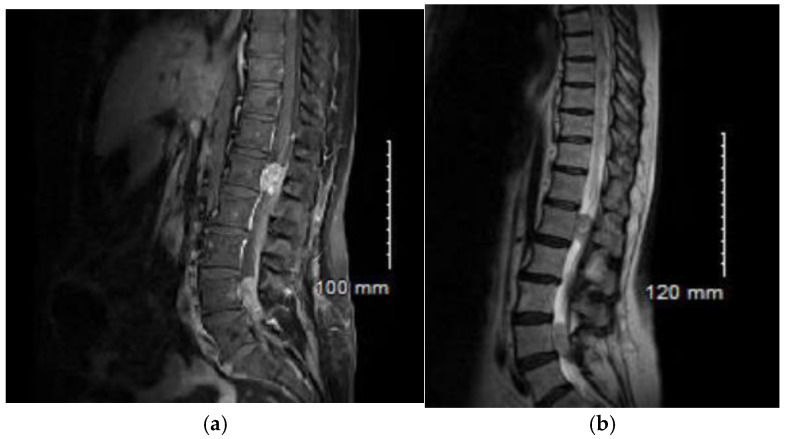
Sagittal magnetic resonance imaging (MRI) showing leptomeningeal carcinomatosis: (**a**) T1-weighted sequence after gadolinium administration; (**b**) T2-weighted FLAIR sequence.

Monitoring continued for 8 months, until the patient experienced progressive neurological disorders, including impaired walking and back pain. A subacute worsening occurred in December 2023, with the onset of symmetrical lower limb motor deficit accompanied by abolished deep tendon reflexes and bilateral Babinski signs. A spinal cord MRI was performed (Figure 4a,b), revealing a T2 hyperintensity of the spinal cord from T1 to the conus medullaris. Additionally, multiple extramedullary contrast-enhancing lesions were noted at levels T2–T3, T7–T8, at the conus medullaris opposite L1, and within the cauda equina roots at L4 and S1. The overall presentation was highly suggestive of leptomeningeal carcinomatosis, with several compressive lesions, notably at the conus medullaris and L4. In this context, the patient received high dose corticotherapy (1 mg/kg) and decompressive radiotherapy targeting levels T12 to S2, with a dose of 20 Gy in 5 fractions.

Unfortunately, the patient’s general condition rapidly deteriorated, marked by the onset of spastic paraplegia, vesicosphincteric dysfunction, and a severely impaired general status. Consequently, exclusive palliative care was initiated in agreement with the patient, who passed away shortly thereafter. No autopsy was performed.

### 3.2. Literature Review

The five included studies, published between 2015 and 2023, comprised three retrospective cohort analyses and two individual case reports. Collectively, they describe clinical outcomes of patients labeled as having POLE-mutated endometrioid endometrial carcinoma (EEC) who experienced recurrence, disease progression, or cancer-related death. However, the molecular annotation across studies was heterogeneous, and in several cohorts, non-canonical POLE variants or multiple-classifier tumors were included, limiting the comparability of results. A detailed summary is provided in Table 1.

Stasenko et al. [10] analyzed a cohort of 451 EECs, including 23 tumors classified as POLE-mutated. Five adverse events were reported within this group: four recurrences and one case of progressive disease. The median time to recurrence was 21 months (95% CI 18.9–23.1), and two patients developed brain metastases. Of note, the cohort included at least one tumor with MSI-high status and a non-canonical POLE variant, raising the possibility of multiple-classifier biology in some cases.

Veneris et al. [11] reported a 49-year-old woman with FIGO stage IVB mixed high-grade carcinoma (serous, clear cell, and endometrioid components) harboring a canonical V411L POLE mutation. After initial chemotherapy, she experienced multiple recurrences, including an abdominal mass, and ultimately received pembrolizumab with a partial response. Given the mixed histology and advanced stage at diagnosis, this case differs from the typical clinical context in which POLE-mutated EECs are usually considered.

McConechy et al. [12] examined 406 endometrial carcinomas and identified 39 POLE-mutated tumors. Three survival events were reported in the POLE group: one recurrence and two disease-specific deaths. This study predated the definition of the canonical POLE hotspot [4], and therefore included some non-canonical variants, which complicates comparison with more recent series.

Mettälä et al. [13] described a fatal recurrence in a 42-year-old immunosuppressed patient with stage IB, grade 1 EEC carrying a canonical A456P mutation. Despite low-risk histologic features, she rapidly developed ovarian and then nodal metastases and died 18 months after diagnosis. Comprehensive genomic profiling revealed a concurrent FGFR2 driver mutation, which may have contributed to the aggressive course.

Billingsley et al. [14] reported a single recurrence among 30 POLE-mutated EECs. The event occurred in a 55-year-old patient with FIGO stage IB, grade 3 carcinoma who developed a pelvic recurrence 23 months after surgery. As with other earlier studies, mutation classification was performed before the current canonical/non-canonical framework was established.

## 4. Discussion

POLE-mutated endometrial carcinomas (POLEmut ECs) are widely recognized for their exceptionally favorable prognosis. This has been consistently demonstrated in retrospective cohorts and in the major randomized PORTEC trials.

This favorable prognosis is thought to be driven, at least in part, by the strong immune activation associated with POLE exonuclease-domain mutations. These mutations create an ultramutated phenotype with abundant neoantigens [15,16], leading to increased CD8+ T-cell infiltration [17] and elevated PD-1/PD-L1 expression [18], features associated with improved anti-tumor response [19].

Against this overwhelmingly positive background, the present case illustrates that exceptionally adverse trajectories can still occur. The combination of a very late recurrence, an atypical metastatic pattern, and a rapidly progressive course prompted us to examine whether similar events had been documented. Our systematic review confirms that such cases, while rare, have indeed been reported. Importantly, many earlier publications predated current molecular standards and included non-canonical POLE variants, tumors with multiple classifiers (particularly POLE/MMRd), or lacked complete molecular annotation. These factors may substantially influence tumor behavior and complicate interpretation of earlier reports.

In contrast, the case presented here involved a canonical hotspot POLE mutation (P286R), with proficient MMR, TP53 wild type, and no additional oncogenic drivers identified, strongly supporting a true canonical POLE-ultramutated profile. The absence of POLE testing on the 2006 primary tumor—unavailable at the time—remains a limitation, but all available data make a misclassification unlikely.These features make this case particularly informative and justify a more detailed examination of three striking aspects: the exceptionally late timing of recurrence, the unusual neuromeningeal dissemination, and the poor prognosis at relapse.

### 4.1. Very Late Recurrence

**Figure 5:**
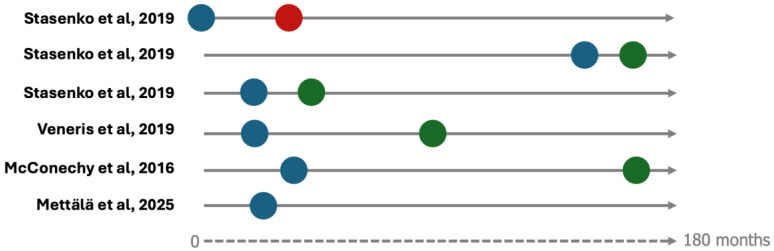
Graphical representation of timelines for reported cases described in Table 1 (when available, [10,11,12,13]). Blue dots indicate time to recurrence; red dots indicate death; green dots indicate last follow-up. Time is represented proportionally in months.

The recurrence observed in our patient occurred 16 years after initial treatment—an extraordinary interval for endometrioid EC. In the general EEC population, over 70% of recurrences occur within 2–3 years [20]. Among the cases identified in our systematic review, Stasenko et al. similarly reported a late recurrence occurring 12 years after initial treatment in a stage IB, grade 3 EEC treated with vaginal brachytherapy [10]. Other reported cases occurred much earlier, with an average progression-free survival of 21 months, consistent with typical recurrence patterns regardless of molecular subgroup [21] (Figure 5).

These very late events raise two important considerations. First, major trials such as PORTEC-1, -2, and -3 had follow-up durations generally below 10 years, making them unable to capture these exceptional delays. Second, current surveillance protocols—which typically involve oncology follow-up for five years followed by primary care without routine imaging—may not detect rare but clinically significant late recurrences in a subgroup otherwise presumed to be cured.

### 4.2. Neuro-Meningeal Tropism

The metastatic pattern observed here—initial cerebral dissemination followed by leptomeningeal carcinomatosis—is highly atypical for EEC, where the vaginal vault is the most common site of recurrence in early-stage disease [21]. Three studies have suggested that when POLEmut EECs recur, they may do so more often at distant sites compared with other molecular subtypes [22,23,24]. Our findings are consistent with this observation, although central nervous system involvement remains extremely rare.

Stasenko et al. reported two cases of brain metastasis in POLEmut EEC [10]. One patient developed an isolated brain lesion 20 months after initial treatment and later required stereotactic radiotherapy and immune checkpoint inhibition upon a second cerebral recurrence. Another patient with FIGO stage IV disease progressed with both breast and brain metastases and died within 33 months. The case we describe adds to this very limited body of evidence suggesting that a neurotropic pattern, although rare, may occur in a small subset of POLEmut tumors.

### 4.3. A Poor Prognosis at Relapse

The rapid progression observed after recurrence in our patient contrasts with the generally favorable salvage outcomes previously reported. In a meta-analysis of 359 POLEmut EECs, McAlpine et al. documented 11 recurrences or progressions, yet 8 of these 11 patients were alive without disease at last follow-up (5.5–14.2 years) [25]. Similarly, in the cohort by Stasenko et al., 3 of 4 patients with recurrence were alive at publication time [10].

In light of these data, the aggressive course observed here highlights that, even within POLEmut tumors, a minority may have biologically distinct and adverse behavior.

### 4.4. Toward Refined Risk Stratification in POLE-Mutated Tumors

Although rare, these cases underscore the need for more individualized risk assessment across POLEmut ECs. Several studies have evaluated potential modifiers of prognosis. A 2021 meta-analysis found that classical prognostic variables—age, grade, LVSI, histotype, adjuvant therapy—did not predict adverse outcomes in POLEmut tumors; only stage retained prognostic significance [25].

On the clinical level, Kolehmainen et al. similarly found no clinical risk factors associated with poorer outcomes within the POLEmut subgroup [26]. At the molecular level, Mettälä et al. identified FGFR2 gain-of-function mutations as potential contributors to recurrence in otherwise low-risk ECs. One patient with a canonical POLE A456P mutation and a concurrent FGFR2 S702L alteration experienced a fatal recurrence within 16 months [13]. Histopathological features may also refine risk stratification. McConechy et al. emphasized that even high-grade or deeply invasive tumors can have excellent outcomes when POLE-mutated [12]. Conversely, He et al. identified the MELF invasion pattern as being associated with a markedly increased risk of recurrence (HR 15.1) [27].

Together, these findings demonstrate that POLE-mutated tumors are not biologically uniform. Rare co-alterations or specific histological features may modify prognosis and could eventually help identify the small subgroup at higher risk.

### 4.5. Limitations

As a single observation, this case cannot support broad conclusions. The review is also limited by the very small number of reported events and by the heterogeneity and incomplete molecular annotation of earlier studies. Nevertheless, it highlights the need for clinical awareness of atypical outcomes, even in a subgroup generally associated with excellent prognosis.

Another limitation is the absence of immunotherapy at the time of leptomeningeal recurrence. Given the high mutational burden of POLEmut tumors, many studies suggest they may respond well to immune checkpoint inhibitors [28,29]. Although immunotherapy was considered, it was ultimately not administered due to the patient’s severely impaired performance status (ECOG 4) and her preference to discontinue active treatments.

Ongoing and recently conducted prospective trials, such as PORTEC-4a [30], will provide more robust data on long-term outcomes and further refine molecular profile-based treatment strategies in this subgroup.

## 5. Conclusions

This case report highlights an exceptional instance of endometrial cancer that, despite carrying a canonical POLE mutation typically associated with an excellent prognosis, evolved in an unexpectedly aggressive manner. Combined with the few similar cases identified in our focused literature review, this observation suggests that prognostic heterogeneity may exist within the POLE-mutated endometrioid carcinoma subgroup. While such events remain exceedingly rare, they underscore that a small minority of POLE-mutated tumors may deviate from the expected favorable trajectory, warranting continued clinical awareness and further investigation.

## Figures and Tables

**Table 1:** Summary of reported cases of recurrence in POLE-mutated endometrioid endometrial carcinoma.

Article	Age (Years)	POLE Mutation	Class of POLE Mutation ^1^	MMR Status ^2^	Stage ^3^	Grade	Adjuvant Therapy	Localization of Recurrence	Treatment at Recurrence	PFS (Months)	Status at Last Contact
[10]	-	P.V411L	Hotspot	pMMR	IV	2	CT ^4^	Brain	Surgery + CT + EBRT ^5^	-	-
	-	P.286R	Hotspot	pMMR	IA	3	IVRT ^6^ + CT	Rectum	Surgery + CT + EBRT	-	-
	-	P.F367V and P.P476S	Non hotspot	dMMR	IB	3	IVRT	Brain	EBRT + immunotherapy	0	Dead at 33 months
	-	P.V411L	Hotspot	dMMR	IB	3	IVRT	Chest wall	Surgery + CT + EBRT	146	Alive at 165 months
	-	P.V411L	Hotspot	pMMR	III	1	IVRT	Vagina, liver	Surgery, CT + EBRT	20	Alive at 42 months
[11]	49	V411L	Hotspot	-	IVB	2	CT	Abdominal mass	CT	21	Alive at 88 months
[12]	52	P286R	Hotspot	-	IB	3	EBRT	-	-	35	Alive with disease at 165 months
[13]	42	A456P	Hotspot	-	IB	1	0	Ovary	Surgery + CT	24	-
[14]	55	-	-	-	IB	3	0	Pelvis	-	-	-

^1^ According to [4]; ^2^ Mismatch Repair (MMR) Status: pMMR (Proficient Mismatch Repair), dMMR (Deficient Mismatch Repair); ^3^ According to FIGO classification, at initial diagnostic; ^4^ Chemotherapy; ^5^ External Beam Radiotherapy; ^6^ Intravaginal Brachytherapy; Unavailable data are represented as “-”.

## Data Availability

The original contributions presented in this study are included in the article. Further inquiries can be directed to the corresponding author.

## Supplementary Materials

The following supporting information can be downloaded at: https://www.mdpi.com/article/10.3390/curroncol33040219/s1, Table S1: PRISMA 2020 Checklist.

## Abbreviations

The following abbreviations are used in this manuscript:

EEC	Endometrioid Endometrial Carcinoma
POLE	Polymerase Epsilon
MMR	Mismatch Repair
MSI	Microsatellite Instability
TCGA	The Cancer Genome Atlas
